# PEX14 binding to *Arabidopsis* PEX5 has differential effects on PTS1 and PTS2 cargo occupancy of the receptor

**DOI:** 10.1016/j.febslet.2014.05.038

**Published:** 2014-06-27

**Authors:** Thomas Lanyon-Hogg, Jacob Hooper, Sarah Gunn, Stuart L. Warriner, Alison Baker

**Affiliations:** a Centre for Plant Sciences, Irene Manton Building, University of Leeds, Leeds LS2 9JT, UK; b School of Chemistry, University of Leeds, Leeds LS2 9JT, UK

**Keywords:** PEX, peroxisome biogenesis, PMP, peroxisomal membrane protein, PTS, peroxisomal targeting signal, FA, fluorescence anisotropy, FI, fluorescence intensity, HRP, horseradish peroxidase, Peroxisome, PEX5, PEX7, PEX14, PTS1, PTS2, Cargo unloading, *Arabidopsis thaliana*

## Abstract

PEX5 acts as a cycling receptor for import of PTS1 proteins into peroxisomes and as a co-receptor for PEX7, the PTS2 receptor, but the mechanism of cargo unloading has remained obscure. Using recombinant protein domains we show PEX5 binding to the PEX14N-terminal domain (PEX14N) has no effect on the affinity of PEX5 for a PTS1 containing peptide. PEX5 can form a complex containing both recombinant PTS1 cargo and endogenous PEX7-thiolase simultaneously but isolation of the complex *via* the PEX14 construct resulted in an absence of thiolase, suggesting a possible role for PEX14 in the unloading of PTS2 cargos.

## Highlights

• The interaction between *Arabidopsis* PEX5 and PEX14N is independent of cargo binding.
• The affinity of a PTS1 peptide for PEX5 is unaffected by PEX14N binding.
• *Arabidopsis* PEX5 complexes PTS1 and PTS2 cargoes.
• PEX5 and 7 co-isolate with PEX14N, but the PTS2 cargo thiolase does not.
• PEX14N does not unload canonical PTS1 cargo peptide in vitro but may play a role in PTS2 release.

## Structured summary of protein interactions

**pMDH1**physically interacts with **PEX5** by pull down (View interaction)

**PEX5C**binds to **PEX14N** by filter binding (View interaction)

**PEX14N**binds to **PEX5C** by pull down (View interaction)

**PEX14N**physically interacts with **PEX7** by pull down (View interaction)

**PEX5**physically interacts with **PEX7** by pull down (View interaction)

**DCI1**physically interacts with **PEX5** by pull down (View interaction)

**PEX5**physically interacts with **thiolase PTS2-cargo** by pull down (View interaction)

**pMDH1**physically interacts with **PEX7** by pull down (View interaction)

**DCI1**physically interacts with **thiolase PTS2-cargo** by pull down (View interaction)

**DCI1**physically interacts with **PEX7** by pull down (View interaction)

**PEX14N**physically interacts with **PEX5** by pull down (View interaction)

## 1 Introduction

Peroxisomal matrix proteins are post-translationally imported from the cytosol *via* a Peroxisomal Targeting Signal (PTS) encoded in the primary structure (reviewed in [1,2]). The majority of matrix proteins possess a PTS1, a C-terminal tripeptide of consensus sequence [S/A/C]-[K/R/H]-[L/M] [3,4], which is recognised by the cytosolic receptor PEX5. Some matrix proteins possess a PTS2, a nonapeptide of consensus sequence [R/K]-[L/V/I]-X_5_-[H/Q]-[L/A] located near the N-terminus [5], which is recognised by the cytosolic receptor PEX7. PEX7 does not function autonomously and import requires co-receptors that vary in a species dependent manner [6]. In *Saccharomyces* *cerevisiae* Pex18p and Pex21p function as the co-receptor, while Pex20p performs this function in other fungi. Recent structural studies show that Pex21p covers the hydrophobic faces of the PTS2 signal and Pex7p to form a stable hydrophobic core [7]. In *Arabidopsis* the co-receptor is PEX5 [8,9], and in mammals a long splice variant of PEX5 (termed PEX5L) [10].

Newly synthesised peroxisomal matrix proteins bind their respective receptors in the cytosol and the complex docks with protein machinery (the ‘importomer’) at the peroxisomal membrane. The receptor docking site consists of the membrane proteins PEX13 and PEX14 (and Pex17p in *S. cerevisiae*) [11]. The N terminus of HsPEX5 binds the N terminus of PEX14 via multiple WX_3_F/Y motifs [12] as well as a newly discovered LVXEF motif [13]. While AtPEX5 contains nine WX_3_F/Y motifs it lacks an obvious counterpart to LVXEF PEX5 inserts into the peroxisomal membrane by a poorly understood mechanism which may involve interactions with membrane lipids [14] as well as with PEX14 [15]. The docking and insertion of PEX5 into the peroxisomal membrane is proposed to be driven solely by favourable thermodynamic interactions [16,17]. The cargo is released into the peroxisome, and the receptor is ubiquitinated by the RING peroxins (PEX2, PEX10, PEX12) [18]. Monoubiquitination of a cysteine residue close to the terminal of PEX5 targets the receptor for release from the peroxisome by the ATPase peroxins PEX1 and PEX6 to begin another round of import [19].

PEX5 and PEX14 have been shown to form a dynamic ligand-gated channel capable of opening to a diameter of 9 nm [15], explaining the ability of peroxisomes to translocate folded proteins without compromising membrane integrity. Binding of PEX5 to its PTS1 cargo has been shown to be unaffected by either interaction of PEX5 with the RING domain of PEX12 [20] or by ubiquitination of PEX5 [21]. The PTS2 protein thiolase has been shown to be released from PEX5 prior to release of the receptor from the peroxisome or ubiquitination of PEX5 [22]. Recent results implicate the redox state of Pex5p and binding Pex8p, an intraperoxisomal protein found only in yeast, in unloading of PTS1 cargo [23]. *Pichia pastoris* Pex5p forms homooligomers through disulfide links at cysteine 10 which results in increased PTS1 binding affinity. Reduction of the disulphide link triggers partial cargo release which is enhanced in the presence of Pex8p. In mammals the PEX5-catalase complex can be dissociated through binding of the N-terminal domain of PEX14 to the N-terminus of PEX5 [24]. Catalase binds through an atypical PTS1 motif [25,26] and additionally has been shown to form interactions with the PEX5N-terminal [24,27]. Disruption of the PEX5–catalase interaction by PEX14 may therefore result from disruption of catalase interactions with the either the PEX5 N-terminal or the TPR domain. *Leishmania donovani* PEX5 shows a decreased affinity for PTS1 cargo in the presence of the PEX14 N-terminus [28]. Attempts to isolate a recombinant *Arabidopsis* PTS1 cargo–PEX5–PEX14 complex have also been unsuccessful, although the corresponding PEX5–PEX14 complex was successfully isolated [29]. This therefore raised the question as to whether the N-terminal domain of PEX14 may function as a general PTS1-cargo unloading species in higher eukaryotes.

In an effort to understand the ordering and function of early stage interactions in the plant peroxisomal import cycle, and to address the question of how cargo is unloaded, we characterised the interactions between PTS1 cargo, PEX5, and the N terminal domain of PEX14 (PEX14N) in assays using defined purified recombinant *Arabidopsis* proteins. To gain insight into the PTS2 pathway we performed pull-downs using a cytosolic extracts of *Arabidopsis* cells as a source of PTS2 pathway components.

## 2 Materials and methods

### 2.1 HRP conjugate binding overlay assays

HRP-maleimide (Sigma) was conjugated to sulfhydryl compounds following the manufacturer’s instructions. Unconjugated sulfhydryls were removed by dialysis into PBS, the HRP-conjugate diluted with glycerol (50% v/v) and stored −20 °C in the dark.

Purified PEX5 (25 pmol) or HRP conjugates (2.5 pmol) were pipetted onto a nitrocellulose membrane, dried, blocked 1 h in 3% BSA PBS-T, then incubated with either peptide-HRP (200 nM) or PEX14N-HRP (50 nM) in blocking buffer for 1 h. Blots were washed 3 times for 10 min in PBS-T. HRP conjugates were visualised using ECL. To assess unloading of HRP-YQSKL from PEX5C by PEX14N, blots were washed 3 times for 10 min in PBS-T, then incubated with PEX14N (0.2 μM) in blocking buffer for 1 h, before washing and visualisation. The process was then repeated with PEX14 N (2 μM) in blocking buffer at 4 °C for 16 h.

### 2.2 Pull-down assays

PEX14N, PEX5C, PEX5, and lissamine-YQSKL (5 μM) in PBS were mixed on ice, adjusted to 1 mL with PBS and incubated with gentle agitation (4 °C, 1 h). The mixture was added to Strep-Tactin resin (500 μL), incubated with gentle agitation (4 °C, 1 h), loaded into a column, drained and washed with PBS (10 × 1.5 mL). Bound complexes were eluted in PBS containing 7.5 mM desthiobiotin (6 × 0.5 mL).

*Arabidopsi*s cytosolic fractions (25 000×*g* supernatant fraction) were prepared from cell cultures as described previously [30] The cytosolic fraction (∼6 mg/mL, 500 μL) was pre-incubated with Ni–NTA (50 μL) resin 4 °C, 1 h, then the unbound fraction added to the recombinant protein binding partner (33 μM) in PBS (50 μL), and incubated with gentle agitation (4 °C, 1 h). Ni–NTA resin (50 μL) was added and the mixture incubated with gentle agitation (4 °C, 1 h), loaded into a column, and washed with PBS (3 × 0.5 mL). Bound complexes were eluted in PBS containing 1 M imidazole (3 × 50 μL).

Immunoblotting was as described [30] using anti-*Arabidopsis* PEX5 [29] at 1:10 000 dilution, anti-*Arabidopsis* PEX7 [31] at 1:1000 dilution, and anti-thiolase [32] at 1:180 000 dilution.

### 2.3 Fluorescence measurement-

Fluorescence studies used an Envision™ 2103 multilabel plate reader (PerkinElmer), Black Optiplate™ F plates (PerkinElmer), excitation filter: 531 (25) nm, dichroic mirror: 555 nm, emission filter: 595 (60) nm. Anisotropy measurements used the equivalent polarised optics and the g-factor was set to 1. For Anisotropy measurements wells were pre-treated with FA buffer (HEPES (20 mM), NaCl (150 mM), pH 7.5) containing 0.32 mg/mL gelatine. To determine the binding affinity of PTS1 peptides, the anisotropy of a dilution series of PEX5C (1 μM–0.04 nM) containing lissamine-YQSKL (100 nM) (final volume 40 μL) was measured before and after addition of 10 μL PEX14N (5 μM) to the wells. To determine the effect of PEX14N concentration on the PTS1–PEX5C complex the anisotropies of solutions containing PEX14N (10 μM–1 nM), PEX5C (100 nM) and lissamine-YQSKL (100 nM) (40 μL final volume) were recorded.

## 3 Results

### 3.1 PEX5 and PEX14N form complexes in the presence or absence of PTS1 cargo

**Fig. 1:**
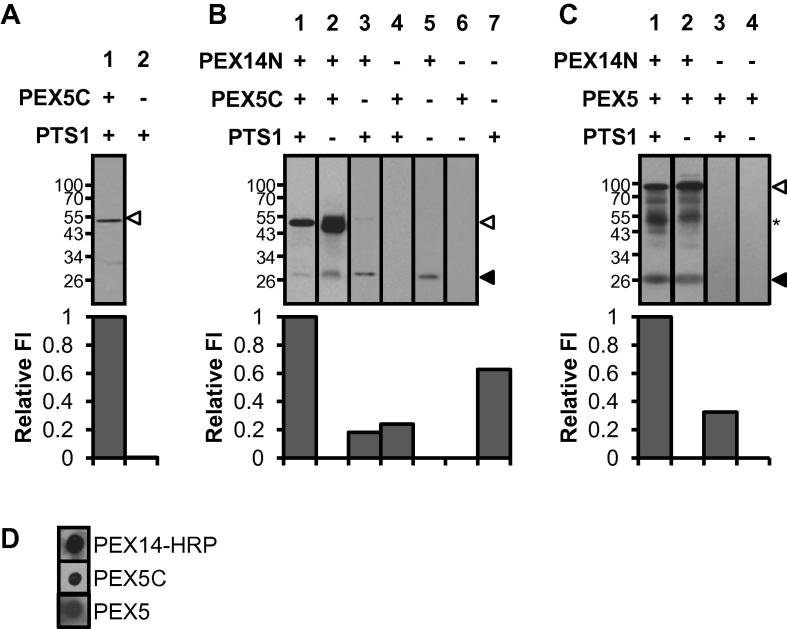
PEX14N binds PEX5 in cargo bound and cargo free form. (A) Lissamine-YQSKL was incubated with Ni–NTA in the presence or absence of PEX5C and bound complexes were eluted. 10 μL of the peak elution fraction (fraction 2) was analysed by anti-polyhistidine immunoblotting to detect PEX5C, 100 μL was analysed by FI at 595 nm to detect Lissamine-YQSKL. PEX5C indicated by open arrow. (B) PEX14N, PEX5C, lissamine-YQSKL were incubated with Strep-Tactin resin as indicated. 10 μL of the peak elution fraction (fraction 2) was analysed by anti-polyhistidine immunoblotting to detect PEX5C and PEX14N, 100 μL was analysed by FI at 595 nm to detect Lissamine-YQSKL. PEX5C indicated by open arrow, PEX14N indicated by solid arrow. (C) PEX14N, PEX5, lissamine-YQSKL were incubated with Strep-Tactin resin as indicated. 10 μL of the peak elution fraction (fraction 2) was analysed by anti-polyhistidine immunoblotting to detect PEX5 and PEX14N, 100 μL was analysed by FI at 595 nm to detect Lissamine-YQSKL. PEX5 indicated by open arrow, PEX14N indicated by solid arrow. (D) PEX5 constructs (2.5 pmol) were pipetted onto nitrocellulose and probed with PEX14-HRP (50 nM).

To determine the ability of PEX5 to form complexes with PEX14N and PTS1 cargo, pull-down experiments were performed utilising purified soluble recombinant constructs (Figs. S1 and S2) and a fluorescently labelled PTS1 peptide. PEX5C bound lissamine-YQSKL (detected using fluorescence) and could be isolated using Ni–NTA resin (Fig. 1A, lane 1). No fluorescence was detected in the absence of PEX5C (Fig. 1A, lane 2). PEX5C contains six of the nine W-X_3_-F/Y motifs that bind the PEX14 N-terminal region. To isolate the ternary complex, a pull-down experiment was performed utilising the StrepII tag of the soluble PEX14N construct (Fig. S1). Controls showed that PEX5C did not bind Strep-Tactin resin (Fig. 1B, lane 6); although minor non-specific interaction between Lissamine-YQSKL (PTS1) and Strep-Tactin was observed (lane 7), which was reduced by the presence of either recombinant protein (Fig. 1B, lanes 3 and 4). A stable ternary PTS1–PEX5C–PEX14N complex was isolated (Fig. 1B, lane 1), and PEX14N could also co-isolate PEX5C in the absence of lissamine-PTS1 cargo (Fig. 1B, lane 2). The pull-down experiments were repeated utilising full-length PEX5. These experiments demonstrated that PEX5 was also capable of forming a stable ternary complex, or binding PEX14N in the absence of PTS1 cargo (Fig. 1C, lanes 1 and 2).

The ability of PEX14N to interact with PEX5 in a cargo-independent manner was confirmed through a binding overlay assay. PEX14N was covalently linked *via* an unique engineered cysteine to HRP-maleimide. PEX5 constructs were pipetted onto a nitrocellulose membrane, which was blocked, probed with 50 nM PEX14N–HRP and detected *via* chemiluminesence. The PEX14N–HRP was capable of forming complexes with both PEX5C and PEX5 in the absence of PTS1 cargo (Fig. 1D).

### 3.2 PEX14N does not release cannonical PTS1 cargo from PEX5C

**Fig. 2:**
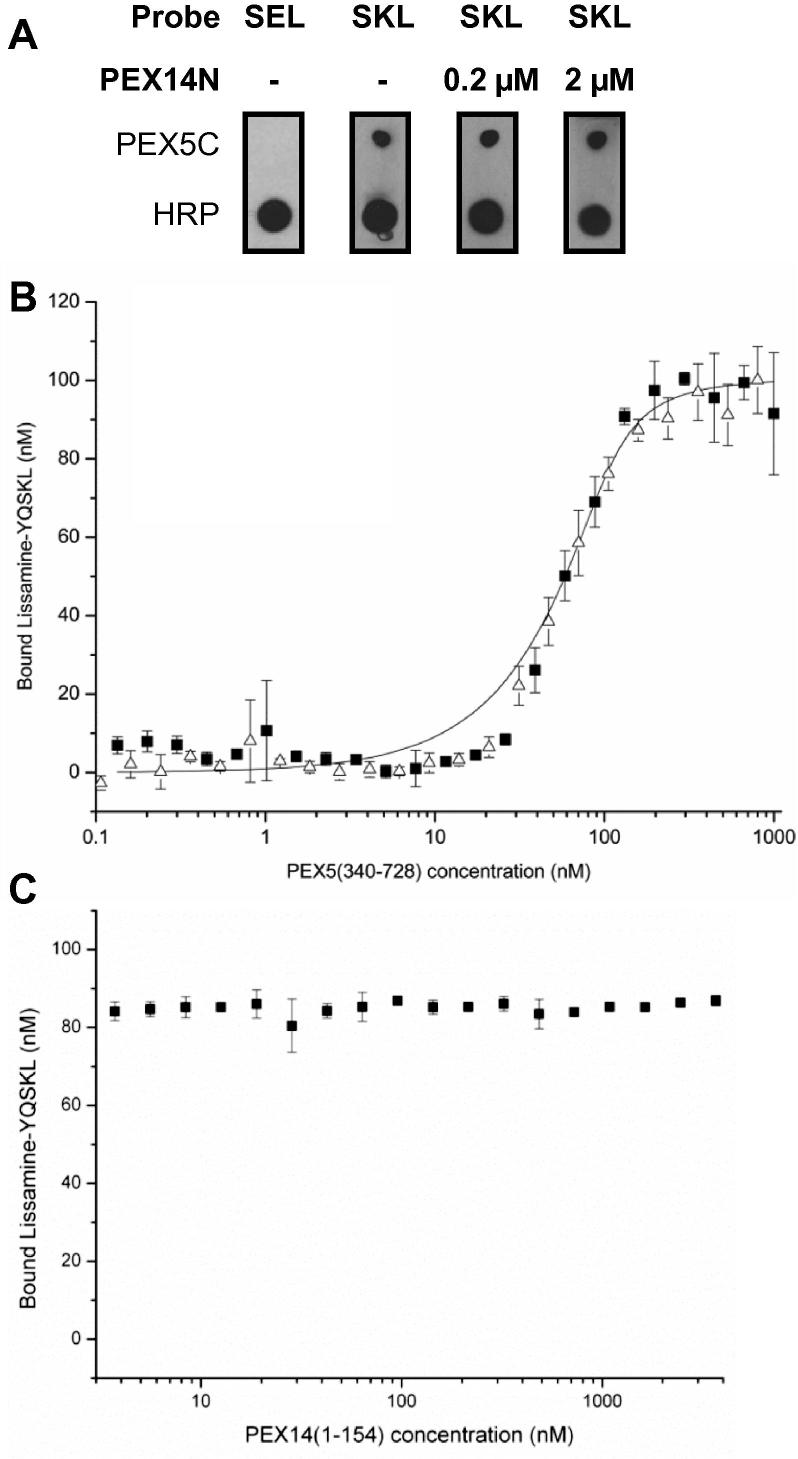
Binding of PEX14N to PEX5C does not release PTS1-cargo. (A) PEX5C and HRP controls were pipetted onto a nitrocellulose membrane and probed with HRP–YQSKL or HRP–YQSEL. HRP–YQSKL probed blot was incubated with indicated concentrations of PEX14N and re-developed. (B) FA measurement of bound lissamine-YQSKL concentration against PEX5C concentration in the presence (black square) or absence (open triangle) of 1 μM PEX14N. Shown binding curve is fitted to titration in the absence of PEX14N, *K*_d_ 8.6 ± 4.0 nM (*R*^2^ = 0.97) [34]. (C) FA measurement of bound lissamine-YQSKL concentration against PEX14N concentration in the presence of 100 nM PEX5C.

To assess whether PEX14N was capable of releasing a generic PTS1 cargo peptide from PEX5, CGGGYQSKL and a non-binding control PTS1 CGGGYQSEL were chemically synthesised and coupled to HRP–maleimide. The peptide–HRP conjugates were used in binding-overlay assays showing that PEX5 bound HRP-SKL, but not HRP–SEL (Fig. 2A). The nitrocellulose bound HRP–SKL–PEX5C complex was incubated for 1 h with 0.2 μM PEX14N, 4 times the concentration used to show PEX14N:PEX5 binding in Fig. 1D, and HRP–SKL was not dissociated. The complex remained even when incubation for a further 16 h with 2 μM PEX14N was performed (Fig. 2A).

A fluorescence anisotropy (FA) binding assay was employed to quantitatively assess the binding interactions [33]. Titration of PEX5C against a constant concentration of lissamine-YQSKL generated a binding curve. Fitting of the data to a 1:1 binding model gave a *K*_d_ for the PEX5C–(lissamine-PTS1) interaction of 8.6 ± 4.0 nM [34]. A constant concentration of PEX14N (1 μM) was then added and the anisotropy measured (Fig. 2B, black squares). Overlay of the datasets indicated identical binding characteristics (*K*_d_ 11.5 ± 3.4 nM) showing that PEX14N does not alter the PEX5C–PTS1 affinity.

To assess if higher molar excesses of PEX14N were required to affect PEX5C–PTS1 interaction, an FA assay was performed titrating PEX14N against a constant concentration of lissamine-YQSKL (100 nM) and PEX5C (100 nM). PEX14N was titrated from 3 μM to 1 nM and the anisotropy measured (Fig. 2C). The anisotropy showed the PTS1–PEX5C binding affinity was unaffected by even a 30-fold molar excess of PEX14N.

### 3.3 PEX5 residues 1–339 are required for co-isolation of PEX7 and thiolase

Attempts to isolate sufficient soluble recombinant *At*PEX7 for binding interaction studies were unsuccessful (data not shown) thus preventing application of the experimental approach presented above to determine the effect of PEX14N on the PEX7–PTS2 interaction. In order to gain insight into the PTS2 pathway, an *Arabidopsis* cytosolic fraction was utilised to allow isolation of PEX7 containing complexes *via* pull-down with hexahistidine tagged recombinant proteins. Prior to use the cytosol was depleted of endogenous nickel binding proteins *via* incubation with Ni–NTA resin. Immunoblotting against PEX5, PEX7 and thiolase (a PTS2 cargo) was used to detect the presence of proteins of interest.

**Fig. 3:**
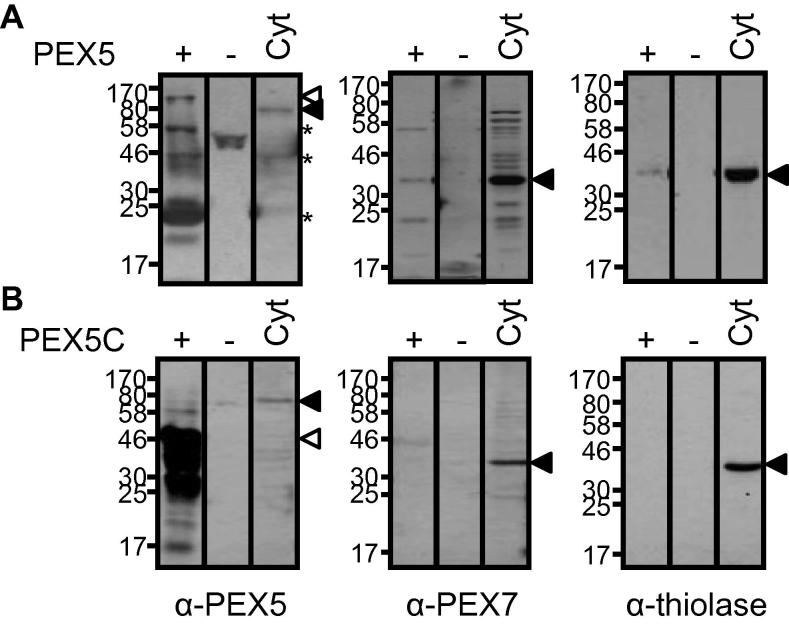
PEX5 binds PEX7 and thiolase but PEX5C does not. *Arabidopsis* cytosol was incubated with Ni–NTA resin in the presence or absence of recombinant PEX5 proteins. The combined elution fractions (10 μL) were analysed by immunoblotting against PEX5, PEX7 or thiolase. In each panel ‘cyt’ is a depleted cytosolic fraction allowing detection of the protein of interest by immunoblotting (positive control), ‘−’ is depleted cytosol to which no recombinant protein is added (negative control) and + is depleted cytosol to which either recombinant PEX5 or PEX5C as indicated was added and recovered by Ni–NTA chromatography. (A) PEX5 pull-down. *Arabidopsis* proteins (closed arrows), His_6_-PEX5 (open arrow), *At*PEX5 degradation products (asterisk). (B) PEX5C pull-down. *Arabidopsis* proteins (closed arrows), His_6_-PEX5C (open arrow).

**Fig. 4:**
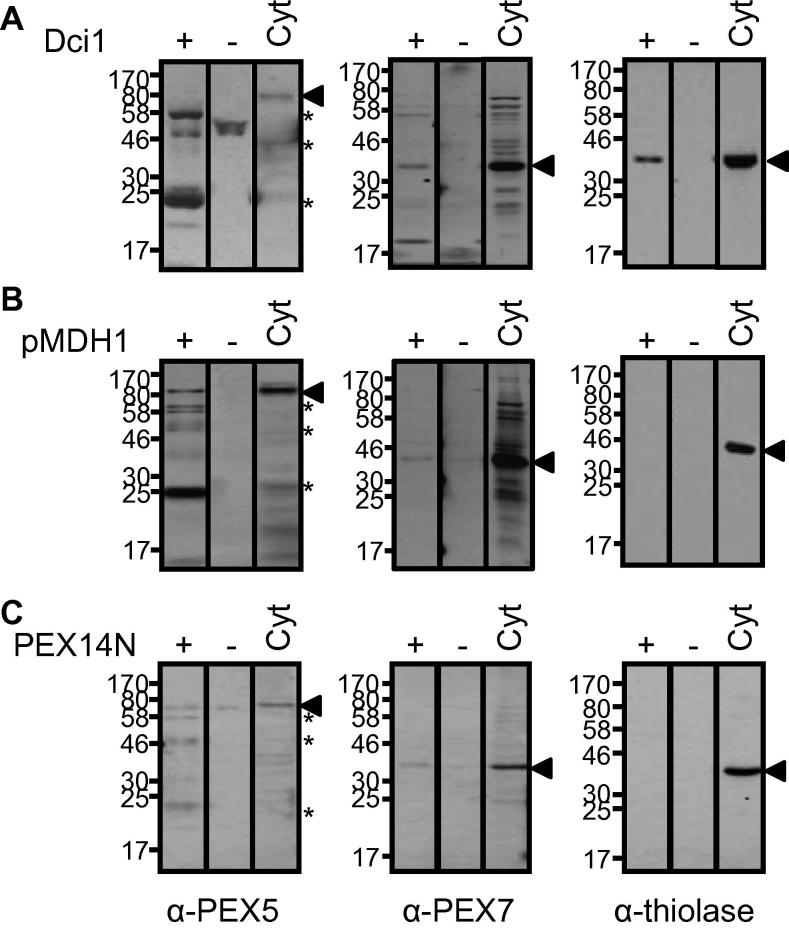
PEX5 can simultaneously bind PTS1 and PEX7–PTS2, however in the presence of PEX14N thiolase PTS2 cargo is not co-isolated. *Arabidopsis* cytosol was incubated with Ni–NTA resin in the presence (+) or absence (−) of recombinant proteins. The combined elution fractions (10 μL) were analysed by immunoblotting against PEX5, PEX7 or thiolase. In each panel ‘cyt’ is a depleted cytosolic fraction allowing detection of the protein of interest by immunoblotting (positive control), ‘−’ is depleted cytosol to which no recombinant protein is added (negative control) and + is depleted cytosol to which recombinant as indicated was added and recovered by Ni–NTA chromatography. (A) Dci1 (PTS1 protein) pull-down. *Arabidopsis* proteins (closed arrows), *At*PEX5 degradation products (asterisk). (B) pMDH1 (PTS2 protein) pull-down. *Arabidopsis* proteins (closed arrows), *At*PEX5 degradation products (asterisk). (C) PEX14N pull-down. *Arabidopsis* proteins (closed arrows), *At*PEX5 degradation products (asterisk).

Pull-down from depleted cytosolic fractions using the recombinant PEX5 construct isolated PEX7 and thiolase PTS2-cargo (Fig. 3A, anti-PEX7 and anti-thiolase panels). The PEX5 N-terminal is sensitive to proteolysis [23,35–37] and during pull down experiments some degradation occurred which was detected as multiple bands by the anti-PEX5 antibody raised against the N-terminal region [29] (Figs. 3 and 4, anti-PEX5 panel). The PEX5C terminal construct lacks residues 314–334 which are required for function as the PEX7 co-receptor [9]. As predicted, pull-down from the depleted cytosol with recombinant PEX5C did not co-isolate either PEX7 or thiolase cargo (Fig. 3B, anti-PEX7 and anti-thiolase panels).

### 3.4 Recombinant PTS1 and PTS2 cargo-proteins, or PEX14N, co-isolate cytosolic PEX5 and PEX7

Δ^3,5^,Δ^2,4^-Dienoyl-coenzyme A isomerase (DCI1) is targeted to the peroxisome *via* a PTS1 sequence [38]. Pull-down from the cytosolic fraction using recombinant DCI1 co-isolated PEX7 and thiolase PTS2-cargo, along with the degradation pattern of PEX5 (Fig. 4A) Isolation of an import complex containing both DCI1 and thiolase cargos indicates that the PEX5 receptor is capable of functioning in both PTS1 and PTS2 pathways simultaneously, either by directly binding both cargoes or by forming mixed PEX5 oligomers containing both cargoes Peroxisomal NAD^+^-malate dehydrogenase (pMDH1) is targeted to the peroxisome *via* a PTS2 sequence [39]. Pull-down from the depleted cytosolic fraction using recombinant pMDH1 co-isolated PEX7 but not thiolase, along with the degradation pattern of PEX5 (Fig. 4B). The lack of thiolase co-isolation in this complex presumably results from the recombinant pMDH1 out-competing thiolase for PEX7 binding. Utilisation of the recombinant PEX14N construct for pull-down from cytosolic fraction co-isolated PEX7 and the degradation pattern of PEX5, but not thiolase PTS2 cargo-protein (Fig. 4C). This result is representative of five independent experiments.

## 4 Discussion

In this study the formation of ternary complexes representative of different steps of receptor recognition and docking was studied using recombinant *Arabidopsis* proteins and domains.

Binding of PTS1-cargo to PEX5 is required for initiation of an import cycle [40], and it has been proposed that a conformational shift in the PEX5 N-terminal region upon PTS1-cargo binding activates PEX14 binding [41]. However, pull-down and binding-overlay studies presented here suggest *Arabidopsis* PEX5 can interact with PEX14 in a PTS1-cargo independent manner. Our observations are in agreement with other studies showing the PEX5–PEX14 interaction can occur in the absence of PTS1-cargo [29,42], and structural studies indicating no major structural rearrangements in the PEX5 N-terminus upon PTS1-cargo binding [43]. Cargo-binding dependent initiation of an import cycle is therefore not mediated through initiation of the PEX5–PEX14 interaction. Given the high affinity of many PTS1–PEX5 interactions which can be low nanomolar, [44], similar to that reported here for AtPEX5C and lissamine YQSKL, the majority of cytosolic PEX5 will exist in cargo loaded state meaning that cargo-free PEX14 complexes are unlikely to form in vivo. Import may be initiated through a PTS1-binding induced conformational shift in the TPR domain [45] which facilitates opening of the PEX5–PEX14 transient pore [15].

Recent studies demonstrate that docking complex components have additional functions [22,24,46]. Binding of the PEX14 N-terminal to the most C-terminal W-X_3_-F/Y motifs has been shown to trigger unloading of the atypical PTS1-cargo catalase from PEX5 [24]. In light of this evidence and the previously reported inability to isolate a PTS1 cargo with an *Arabidopsis* PEX5–PEX14 N-terminus complex [29], the ability of PEX14N to disrupt the PTS1–PEX5 interaction was examined. The 1:1:1 molar ratio of components in pull-down assays that allowed successful isolation of the ternary complex may have contained insufficient PEX14N to trigger unloading due to the higher PEX14N affinity of the N-terminal W-X_3_-F/Y motif of PEX5 [12]. However, in both binding-overlay and FA assays even substantial molar excesses of PEX41N did not trigger release of PTS1-cargo from PEX5 or affect the binding affinity. However, this system differs in important ways from the in vivo situation where interactions take place within the context of the membrane and complete cargo proteins may make additional interactions with PEX5 that could, as in the case of catalase, be PEX14 sensitive. Nevertheless our data indicate that in vitro PEX14N binding to PEX5 is not sufficient to disrupt interactions between PEX5 and the key binding residues of a canonical PTS1 peptide.

Pull-down experiments from cytosolic extracts using recombinant PEX5 were in agreement with in vivo data demonstrating that PEX5 amino acids 314–334 are required for PEX7 interaction [9] as PEX7 co-isolated with full length PEX5 but not PEX5C. Attempts to detect a range of PTS1 cargo-proteins were not successful (data not shown), presumably due to the higher abundance of PTS1 proteins causing individual PTS1 proteins to be below the detection limit in the complex. The less-common utilisation of PTS2 signals results in lower competition for receptor binding; additionally PTS2 proteins released from peroxisomes during preparation of the cytosolic fraction can have had their PTS2-sequences proteolytically cleaved within the peroxisome [22]. Isolation of the preimport complex *via* recombinant PTS1-cargo co-isolated PEX7 and thiolase PTS2 cargo, indicating that PEX5 is capable of binding both PTS1- and PTS2-cargo, or PTS1 and a PEX7/PTS2 loaded second molecule of PEX5 simultaneously.

Interestingly, we were not successful in isolating thiolase using recombinant PEX14N, although the isolated complex contained PEX7. The absence of thiolase in this complex can be accounted for by two hypotheses; firstly PTS2 cargo may be unloaded from the complex through binding with PEX14N, or secondly PEX14N may show a higher affinity for PEX5–PEX7 than for PEX5–PEX7–PTS2. Without recombinant AtPEX7 it is not possible to unambiguously distinguish between these possibilities, however in vitro import assays demonstrate that interaction with docking complex components is sufficient for unloading of thiolase into the peroxisome [22]. This is compatible with the hypothesis that thiolase is dissociated from the import complex through the binding of PEX14. In addition, PEX5L–(PEX7–PTS2) complexes have been shown to be more stable in CHO *pex14* cell lines [47]. Both catalase and thiolase (directly or *via* PEX7) require interactions with the PEX5 N-terminal region for import, which is also the PEX5 region containing the PEX14N binding motifs. Structural data from yeast shows the PEX7 co-receptor, Pex21p, forms a lid over the bound PTS2 sequence [7]. Conformational shifts in the co-receptor upon PEX14N binding may disrupt these favourable interactions with the PTS2 signal. Interestingly the 37 amino acid insert allowing mammalian PEX5L to function as a PEX7 co-receptor also contains a PEX14N binding motif.

Our data are compatible with a growing body of evidence supporting cargo release occurring at the stage of receptor interaction with the docking complex [22,24] but our data suggest the N-terminal domain of PEX14 is not sufficient, at least in vitro, for cannonical PTS1 cargo unloading. The obvious other candidate for involvement in unloading of canonical PTS1-cargos at this stage of import would therefore be PEX13, the second component of the docking complex. Pull-down experiments have previously demonstrated PEX13 can co-isolate PEX5 but not a PEX5–PTS1 complex [47], and in vivo studies indicate that efficient PTS1 import requires PEX5–PEX13 interaction [48,49]. The differential interaction of PEX13 and PEX14 with receptors may therefore allow them to function as both docking site and cargo unloading site.

## Appendix A Supplementary data

Supplementary Fig. 1 Schematic diagram of recombinant proteins used in this study. Scale bar denotes residue numbers. (A) *Arabidopsis* PEX5. (B) PEX5 (1–728), termed PEX5. (C) PEX5 (340–728), termed PEX5C. (D) *Arabidopsis* PEX14. (E) PEX14 (1–154), termed PEX14N. Numbered boxes indicate W-X_3_-F/Y motifs for PEX14 binding. Hashed white box indicates PEX7 binding region. TRP indicates tetratricopeptide repeat domain for canonical PTS1 peptide binding. TM indicates putative transmembrane domain. Coiled-coil indicates coiled-coil domain. Lined white box indicates unique engineered cysteine residue. Affinity tags not show to scale.

Supplementary Fig. 2 Purity of recombinant proteins evaluated by SDS PAGE and Coomassie Blue staining. Recombinant proteins were expressed in *E. coli* and purified *via* IMAC (see Materials and methods). Proteins were separated *via* SDS–PAGE and analysed by Coomassie Blue staining (left) and anti-polyhistidine immunoblotting (right).

Supplementary data 3 This document contains supplementary materials.

## References

[1] Lanyon-Hogg T., Warriner S.L., Baker A. (2010). Getting a camel through the eye of a needle: the import of folded proteins by peroxisomes. Biol. Cell.

[2] Rucktaschel R., Girzalsky W., Erdmann R. (2011). Protein import machineries of peroxisomes. Biochim. Biophys. Acta.

[3] Lametschwandtner G., Brocard C., Fransen M., Van Veldhoven P., Berger J., Hartig A. (1998). The difference in recognition of terminal tripeptides as peroxisomal targeting signal 1 between yeast and human is due to different affinities of their receptor Pex5p to the cognate signal and to residues adjacent to it. J. Biol. Chem..

[4] Reumann S., Quan S., Aung K., Yang P.F., Manandhar-Shrestha K., Holbrook D., Linka N., Switzenberg R., Wilkerson C.G., Weber A.P.M., Olsen L.J., Hu J.P. (2009). In-depth proteome analysis of Arabidopsis leaf peroxisomes combined with in vivo subcellular targeting verification indicates novel metabolic and regulatory functions of peroxisomes. Plant Physiol..

[5] Rachubinski R.A., Subramani S. (1995). How proteins penetrate peroxisomes. Cell.

[6] Brown L.A., Baker A. (2008). Shuttles and cycles: transport of proteins into the peroxisome matrix. Mol. Membr. Biol..

[7] Pan D.Q., Nakatsu T., Kato H. (2013). Crystal structure of peroxisomal targeting signal-2 bound to its receptor complex Pex7p–Pex21p. Nat. Struct. Mol. Biol..

[8] Hayashi M., Yagi M., Nito K., Kamada T., Nishimura M. (2005). Differential contribution of two peroxisomal protein receptors to the maintenance of peroxisomal functions in Arabidopsis. J. Biol. Chem..

[9] Woodward A.W., Bartel B. (2005). The Arabidopsis peroxisomal targeting signal type 2 receptor PEX7 is necessary for peroxisome function and dependent on PEX5. Mol. Biol. Cell.

[10] Otera H., Harano T., Honsho M., Ghaedi K., Mukai S., Tanaka A., Kawai A., Shimizu N., Fujiki Y. (2000). The mammalian peroxin Pex5pL, the longer isoform of the mobile peroxisome targeting signal (PTS) type 1 transporter, translocates the Pex7p.PTS2 protein complex into peroxisomes via its initial docking site, Pex14p. J. Biol. Chem..

[11] Agne B., Meindl N.M., Niederhoff K., Einwachter H., Rehling P., Sickmann A., Meyer H.E., Girzalsky W., Kunau W.H. (2003). Pex8p: an intraperoxisomal organizer of the peroxisomal import machinery. Mol. Cell.

[12] Saidowsky J., Dodt G., Kirchberg K., Wegner A., Nastainczyk W., Kunau W.H., Schliebs W. (2001). The di-aromatic pentapeptide repeats of the human peroxisome import receptor PEX5 are separate high affinity binding sites for the peroxisomal membrane protein PEX14. J. Biol. Chem..

[13] Neuhaus A., Kooshapur H., Wolf J., Meyer N.H., Madl T., Saidowsky J., Hambrunch E., Lazam A., Jung M., Settler M., Schliebs W., Erdman R. (2014). A novel PEX14 protein-interacting site of human PEX5 is critical for matrix protein import into peroxisomes. J. Biol. Chem..

[14] Kerssen D., Hambruch E., Klaas W., Platta H.W., de Kruijff B., Erdmann R., Kunau W.H., Schliebs W. (2006). Membrane association of the cycling peroxisome import receptor Pex5p. J. Biol. Chem..

[15] Meinecke M., Cizmowski C., Schliebs W., Kruger V., Beck S., Wagner R., Erdmann R. (2010). The peroxisomal importomer constitutes a large and highly dynamic pore. Nat. Cell Biol..

[16] Oliveira M.E., Gouveia A.M., Pinto R.A., Sa-Miranda C., Azevedo J.E. (2003). The energetics of Pex5p-mediated peroxisomal protein import. J. Biol. Chem..

[17] Miyata N., Fujiki Y. (2005). Shuttling mechanism of peroxisome targeting signal type 1 receptor Pex5: ATP-independent import and ATP-dependent export. Mol. Cell. Biol..

[18] Williams C., van den Berg M., Geers E., Distel B. (2008). Pex10p functions as an E3 ligase for the Ubc4p-dependent ubiquitination of Pex5p. Biochem. Biophys. Res. Commun..

[19] Kiel J., Emmrich K., Meyer H.E., Kunau W.H. (2005). Ubiquitination of the peroxisomal targeting signal type 1 receptor, Pex5p, suggests the presence of a quality control mechanism during peroxisomal matrix protein import. J. Biol. Chem..

[20] Harper C.C., Berg J.M., Gould S.J. (2003). PEX5 binds the PTS1 independently of Hsp70 and the peroxin PEX12. J. Biol. Chem..

[21] Grou C.P., Carvalho A.F., Pinto M.P., Huybrechts S.J., Sa-Miranda C., Fransen M., Azevedo J.E. (2009). Properties of the ubiquitin-Pex5p thiol ester conjugate. J. Biol. Chem..

[22] Alencastre I.S., Rodrigues T.A., Grou C.P., Fransen M., Sá-Miranda C., Azevedo J.E. (2009). Mapping the cargo protein membrane translocation step into the PEX5 cycling pathway. J. Biol. Chem..

[23] Ma C., Hagstrom D., Polley S.G., Subramani S. (2013). Redox-regulated cargo binding and release by the peroxisomal targeting signal receptor, Pex5. J. Biol. Chem..

[24] Freitas M.O., Francisco T., Rodrigues T.A., Alencastre I.S., Pinto M.P., Grou C.P., Carvalho A.F., Fransen M., Sa-Miranda C., Azevedo J.E. (2011). PEX5 protein binds monomeric catalase blocking its tetramerization and releases it upon binding the N-terminal domain of PEX14. J. Biol. Chem..

[25] Williams C., Bener Aksam E., Gunkel K., Veenhuis M., van der Klei I.J. (2012). The relevance of the non-canonical PTS1 of peroxisomal catalase. Biochim. Biophys. Acta.

[26] Purdue P.E., Lazarow P.B. (1996). Targeting of human catalase to peroxisomes is dependent upon a novel COOH-terminal peroxisomal targeting sequence. J. Cell Biol..

[27] Oshima Y., Kamigaki A., Nakamori C., Mano S., Hayashi M., Nishimura M., Esaka M. (2008). Plant catalase is imported into peroxisomes by pex5p but is distinct from typical PTS1 import. Plant Cell Physiol..

[28] Madrid K.P., De Crescenzo G., Wang S.W., Jardim A. (2004). Modulation of the Leishmania donovani peroxin 5 quaternary structure by peroxisomal targeting signal 1 ligands. Mol. Cell. Biol..

[29] Nito K., Hayashi M., Nishimura M. (2002). Direct interaction and determination of binding domains among peroxisomal import factors in Arabidopsis thaliana. Plant Cell Physiol..

[30] Mitsuya S., El-Shami M., Sparkes I.A., Charlton W.L., Lousa Cde M., Johnson B., Baker A. (2010). Salt stress causes peroxisome proliferation, but inducing peroxisome proliferation does not improve NaCl tolerance in Arabidopsis thaliana. PloS One.

[31] Brown L.A., O’Leary-Steele C., Brookes P., Armitage L., Kepinski S., Warriner S.L., Baker A. (2011). A small molecule with differential effects on the PTS1 and PTS2 peroxisome matrix import pathways. Plant J..

[32] Germain V., Rylott E.L., Larson T.R., Sherson S.M., Bechtold N., Carde J.P., Bryce J.H., Graham I.A., Smith S.M. (2001). Requirement for 3-ketoacyl-CoA thiolase-2 in peroxisome development, fatty acid beta-oxidation and breakdown of triacylglycerol in lipid bodies of Arabidopsis seedlings. Plant J..

[33] Gatto G.J., Geisbrecht B.V., Gould S.J., Berg J.M. (2000). Peroxisomal targeting signal-1 recognition by the TPR domains of human PEX5. Nat. Struct. Biol..

[34] We reproducibly observe a slight deviation from theoretical single site binding model curves at lower PEX5 concentrations in these anisotropy measurements suggesting some more subtle equilibria, perhaps involving PEX5 multimers is present, however these minor differences do not affect the analysis presented herein.

[35] Costa-Rodrigues J., Carvalho A.F., Fransen M., Hambruch E., Schliebs W., Sa-Miranda C., Azevedo J.E. (2005). Pex5p, the peroxisomal cycling receptor, is a monomeric non-globular protein. J. Biol. Chem..

[36] Carvalho A.F., Costa-Rodrigues J., Correia I., Pessoa J.C., Faria T.Q., Martins C.L., Fransen M., Sa-Miranda C., Azevedo J.E. (2006). The N-terminal half of the peroxisomal cycling receptor Pex5p is a natively unfolded domain. J. Mol. Biol..

[37] Moscicka K.B., Klompmaker S.H., Wang D.Y., van der Klei I.J., Boekema E.J. (2007). The Hansenula polymorpha peroxisomal targeting signal 1 receptor, Pex5p, functions as a tetramer. FEBS Lett..

[38] Goepfert S., Vidoudez C., Rezzonico E., Hiltunen J.K., Poirier Y. (2005). Molecular identification and characterization of the Arabidopsis Delta(3,5), Delta (2,4)-dienoyl-coenzyme A isomerase, a peroxisomal enzyme participating in the beta-oxidation cycle of unsaturated fatty acids. Plant Physiol..

[39] Pracharoenwattana I., Cornah J.E., Smith S.M. (2007). Arabidopsis peroxisomal malate dehydrogenase functions in beta-oxidation but not in the glyoxylate cycle. Plant J..

[40] Gouveia A.M., Guimaraes C.P., Oliveira M.E., Sa-Miranda C., Azevedo J.E. (2003). Insertion of Pex5p into the peroxisomal membrane is cargo protein-dependent. J. Biol. Chem..

[41] Harano T., Nose S., Uezu R., Shimizu N., Fujiki Y. (2001). Hsp70 regulates the interaction between the peroxisome targeting signal type 1 (PTS1)-receptor Pex5p and PTS1. Biochem. J..

[42] Urquhart A.J., Kennedy D., Gould S.J., Crane D.I. (2000). Interaction of Pex5p, the type 1 peroxisome targeting signal receptor, with the peroxisomal membrane proteins Pex14p and Pex13p. J. Biol. Chem..

[43] Shiozawa K., Konarev P.V., Neufeld C., Wilmanns M., Svergun D.I. (2009). Solution structure of human Pex5.Pex14.PTS1 protein complexes obtained by small angle X-ray scattering. J. Biol. Chem..

[44] Ghosh D., Berg J.M. (2010). A proteome-wide perspective on peroxisome targeting signal 1(PTS1)-Pex5p affinities. J. Am. Chem. Soc..

[45] Stanley W.A., Wilmanns M. (2006). Dynamic architecture of the peroxisomal import receptor Pex5p. Biochim. Biophys. Acta.

[46] Bharti P., Schliebs W., Schievelbusch T., Neuhaus A., David C., Kock K., Herrmann C., Meyer H.E., Wiese S., Warscheid B., Theiss C., Erdmann R. (2011). PEX14 is required for microtubule-based peroxisome motility in human cells. J. Cell Sci..

[47] Mukai S., Fujiki Y. (2006). Molecular mechanisms of import of peroxisome-targeting signal type 2 (PTS2) proteins by PTS2 receptor pex7p and PTS1 receptor Pex5pL. J. Biol. Chem..

[48] Pires J.R., Hong X.J., Brockmann C., Volkmer-Engert R., Schneider-Mergener J., Oschkinat H., Erdmann R. (2003). The ScPex13p SH3 domain exposes two distinct binding sites for Pex5p and Pex14p. J. Mol. Biol..

[49] Schell-Steven A., Stein K., Amoros M., Landgraf C., Volkmer-Engert R., Rottensteiner H., Erdmann R. (2005). Identification of a novel, intraperoxisomal Pex14-binding site in Pex13: association of Pex13 with the docking complex is essential for peroxisomal matrix protein import. Mol. Cell. Biol..

