# Supplementary material for: PEX14 binding to Arabidopsis PEX5 has differential effects on PTS1 and PTS2 cargo occupancy of the receptor
Source: FEBS Lett. 2014 Jun 27;588(14):2223–9. doi: 10.1016/j.febslet.2014.05.038 (PMC4065332; doi:10.1016/j.febslet.2014.05.038)
Supplement: Supplementary Fig. 1 — Schematic diagram of recombinant proteins used in this study. Scale bar denotes residue numbers. (A) Arabidopsis PEX5. (B) PEX5 (1–728), termed PEX5. (C) PEX5 (340–728), termed PEX5C. (D) Arabidopsis PEX14. (E) PEX14 (1–154), termed PEX14N. Numbered boxes indicate W-X3-F/Y motifs for PEX14 binding. Hashed white box indicates PEX7 binding region. TRP indicates tetratricopeptide repeat domain for canonical PTS1 peptide binding. TM indicates putative transmembrane domain. Coiled-coil indicates coiled-coil domain. Lined white box indicates unique engineered cysteine residue. Affinity tags not show to scale. [file mmc1.pptx]

## Slide 1
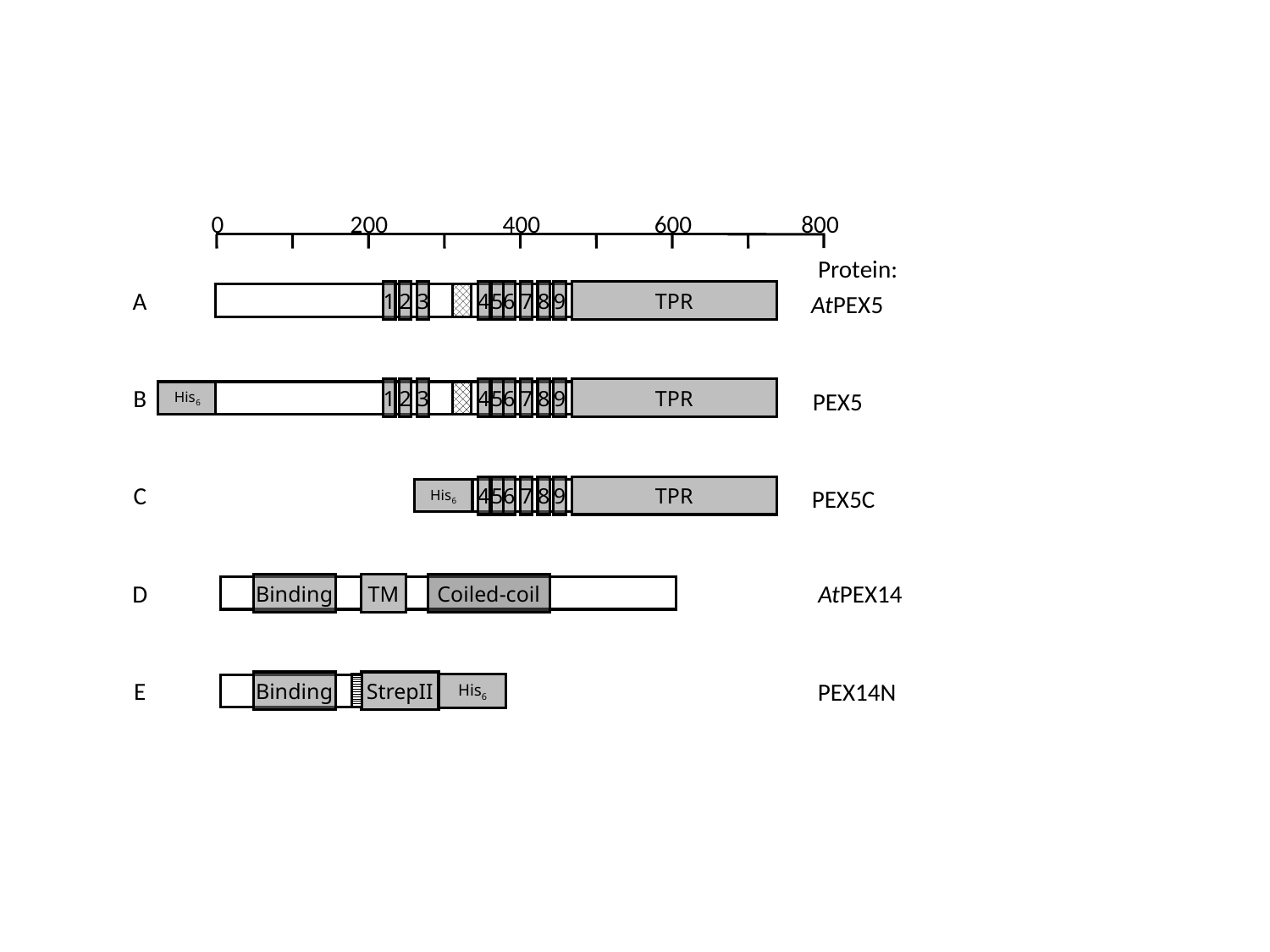

0
200
400
600
800
Protein:
A
AtPEX5
TPR
1
2
3
4
5
6
7
8
9
B
PEX5
His6
TPR
1
2
3
4
5
6
7
8
9
C
PEX5C
His6
TPR
4
5
6
7
8
9
D
AtPEX14
Binding
TM
Coiled-coil
E
PEX14N
Binding
StrepII
His6
