# Supplementary figures and images for: PEX14 binding to Arabidopsis PEX5 has differential effects on PTS1 and PTS2 cargo occupancy of the receptor
Source: FEBS Lett. 2014 Jun 27;588(14):2223–9. doi: 10.1016/j.febslet.2014.05.038 (PMC4065332; doi:10.1016/j.febslet.2014.05.038)

## Slide 1
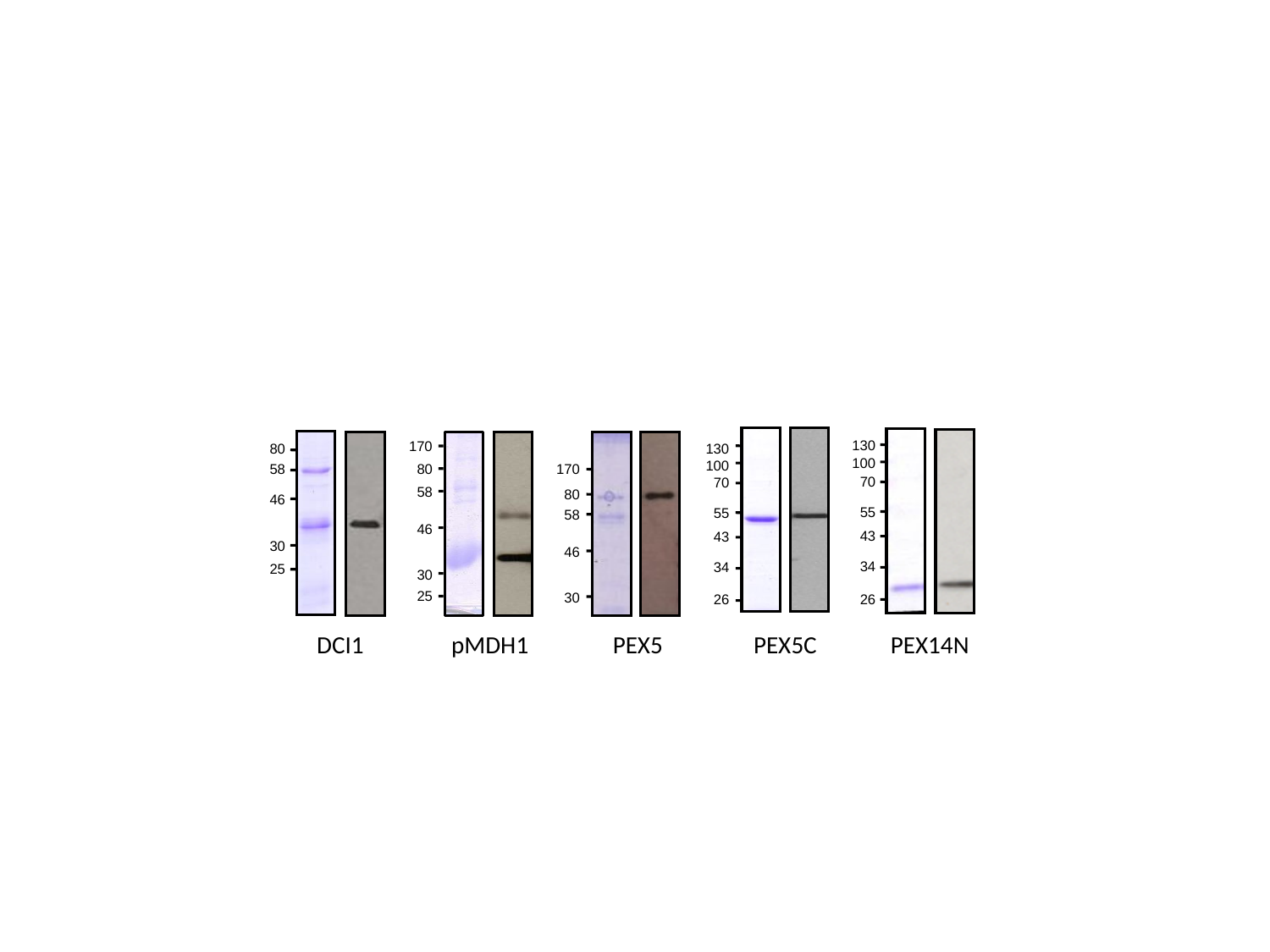

130
170
130
80
100
100
170
58
80
70
70
58
80
46
55
55
58
46
43
43
30
46
34
34
25
30
25
30
26
26
DCI1
pMDH1
PEX5
PEX5C
PEX14N

Supplement: Supplementary Fig. 2 — Purity of recombinant proteins evaluated by SDS PAGE and Coomassie Blue staining. Recombinant proteins were expressed in E. coli and purified via IMAC (see Materials and methods). Proteins were separated via SDS–PAGE and analysed by Coomassie Blue staining (left) and anti-polyhistidine immunoblotting (right). [file mmc2.pptx]
