# Supplementary material for: PEX14 binding to Arabidopsis PEX5 has differential effects on PTS1 and PTS2 cargo occupancy of the receptor
Source: FEBS Lett. 2014 Jun 27;588(14):2223–9. doi: 10.1016/j.febslet.2014.05.038 (PMC4065332; doi:10.1016/j.febslet.2014.05.038)
Supplement: Supplementary data 3 — This document contains supplementary materials. [file mmc3.pdf]

Lanyon-Hogg et al Supplementary Information.

Supplementary methods.

Plasmid constructs

PEX5(1-728) (termed PEX5) and PEX5(340-728) (termed PEX5C) were amplified from the Yeast 2 Hybrid vector pGADT7/Rec PEX5 [1] using PCR primers 5’-GCGGATCCTCACAGCGGGAATTCTTTCTG-3’ (reverse) and 5’-CCCCCATATGCAAGCTTCAGCCCCCG-3’ (PEX5C forward) or 5’-CCCCCATATGGCGATGAGAGACCTTG-3’ (PEX5 forward) and the PCR products cloned into the pGEMTeasy vector. The amplified fragment was digested with *Nde*I and *Bam*HI and cloned into *Nde*I/*Bam*HI digested pET28b vector.

PEX14(1-154) (termed PEX14N) was amplified using PCR primers 5’-CCCCATGGCAACTC ATCAGCAAAC-3’ (forward) and 5’-AACTCGAG*TTTTTCGAACTGCGGGTGGCTCCAGCA*ATGGTACCAGCGAAACCG-3’ (reverse). The PCR product was cloned into pET28b *via* the *Nho*I and *Xho*I sites

Recombinant Proteins-

The His_6-*At*DCI1 and pMDH1-His_6 construction and purification are described in [38] and [39] respectively

PEX5C and PEX5 were expressed in the BL21-Codon Plus (DE3) RIL and lysed *via* sonication in lysis buffer (NaH_2PO_4 (50 mM), NaCl (300 mM), Glycerol (15 % v/v), pH 8.0 β-metacapethanol (10 mM). PEX5C was expressed at 28 ^oC under autoinduction conditions [2], and purified using Co-NTA resin (washes with lysis buffer containing 20 mM imidazole and eluted in lysis buffer containing 200 mM imidazole). Cells expressing PEX5 were grown to an OD_{600} of 0.4 to 0.6, heat shocked for 20 min at 45 ^oC, then cooled to 18 ^oC. Expression was induced by addition of IPTG (1 mM) and the cells grown for a further 18 h at 18 ^oC. PEX5 was purified using Ni-NTA resin (washes with lysis buffer containing 20, 40 and 60 mM imidazole and EDTA free protease inhibitors and eluted with lysis buffer containing 250 mM imidazole).

PEX14N was expressed in BL21 STAR™ (DE3)/pRARE2 under autoinduction conditions at 28 ^{o}C. Cells were lysed *via* sonication in lysis buffer and purified using Co-NTA resin (washes with lysis buffer containing 5 mM β-mercaptoethanol and 20 mM imidazole and eluted in lysis buffer containing 5 mM β-mercaptoethanol and 200 mM imidazole).

Supplementary references

1. Bonsegna, S., Slocombe, S. P., De Bellis, L. & Baker, A. (2005) AtLACS7 interacts with the TPR domains of the PTS1 receptor PEX5, *Arch Biochem Biophys.* **443**, 74-81.
2. Studier, F. W. (2005) Protein production by auto-induction in high density shaking cultures, *Protein Expr Purif.* **41**, 207-34.

Supplementary figure legends

Figure S1. Schematic diagram of recombinant proteins used in this study.
Scale bar denotes residue numbers. (A) *Arabidopsis* PEX5. (B) PEX5(1-728), termed PEX5. (C) PEX5(340-728), termed PEX5C. (D) *Arabidopsis* PEX14. (E) PEX14(1-154), termed PEX14N. Numbered boxes indicate W-X_3-F/Y motifs for PEX14 binding. Hashed white box indicates PEX7 binding region. TRP indicates tetratricopeptide repeat domain for canonical PTS1 peptide binding. TM indicates putative transmembrane domain. Coiled-coil indicates coiled-coil domain. Lined white box indicates unique engineered cysteine residue. Affinity tags not show to scale.

Figure S2 Purity of recombinant proteins evaluated by SDS PAGE and Coomassie Blue staining.

Recombinant proteins were expressed in *E. coli* and purified *via* IMAC (see Materials and Methods). Proteins were separated *via* SDS-PAGE and analysed by Coomassie Blue staining (left) and anti-polyhistidine immunoblotting (right).
